# Ashwagandha Leaf Derived Withanone Protects Normal Human Cells Against the Toxicity of Methoxyacetic Acid, a Major Industrial Metabolite

**DOI:** 10.1371/journal.pone.0019552

**Published:** 2011-05-04

**Authors:** Didik Priyandoko, Tetsuro Ishii, Sunil C. Kaul, Renu Wadhwa

**Affiliations:** 1 National Institute of Advanced Industrial Science & Technology (AIST), Tsukuba, Ibaraki, Japan; 2 Graduate School of Life & Environmental Sciences, University of Tsukuba, Tsukuba, Ibaraki, Japan; The University of Texas at Austin, United States of America

## Abstract

The present day lifestyle heavily depends on industrial chemicals in the form of agriculture, cosmetics, textiles and medical products. Since the toxicity of the industrial chemicals has been a concern to human health, the need for alternative non-toxic natural products or adjuvants that serve as antidotes are in high demand. We have investigated the effects of Ayurvedic herb Ashwagandha (*Withania somnifera*) leaf extract on methoxyacetic acid (MAA) induced toxicity. MAA is a major metabolite of ester phthalates that are commonly used in industry as gelling, viscosity and stabilizer reagents. We report that the MAA cause premature senescence of normal human cells by mechanisms that involve ROS generation, DNA and mitochondrial damage. Withanone protects cells from MAA-induced toxicity by suppressing the ROS levels, DNA and mitochondrial damage, and induction of cell defense signaling pathways including Nrf2 and proteasomal degradation. These findings warrant further basic and clinical studies that may promote the use of withanone as a health adjuvant in a variety of consumer products where the toxicity has been a concern because of the use of ester phthalates.

## Introduction

Methoxyacetic acid (MAA, CH_3_OCH_2_COOH) is a primary metabolite of ester phthalates, a class of industrial chemicals used in a large variety of consumer products including household products (building materials, plastic objects, textiles, adhesives, paints and deodorants), food and personal care products including agricultural adjuvant, pesticides, cosmetics and perfumes, electronics (coatings, stabilizers and surfactants) and pharmaceuticals (enteric coating of oral pills, viscosity control agents, surfactants and stabilizers). The most widely-used phthalates are the di-2-ethyl hexyl phthalate (DEHP)- a low cost plasticizer used in PVC, benzylbutylphthalate (BBzP)- used in the manufacture of foamed PVC used as a flooring material, the diisodecyl phthalate (DIDP)-viscosity controlling reagent, the diisononyl phthalate (DINP) and small phthalates-used as solvents in perfumes and pesticides. Globally, more than 18 billion pounds of ester phthalates are used annually and toxicity of these chemicals occurs through ingestion, inhalation, intravenous injection and dermal exposure on a daily basis. In the United States and Canada, DEHP is no longer used to manufacture children's products intended for mouthing, such as pacifiers, but it is still used in larger toys. DEHP is approved for use in medical devices such as surgical vinyl gloves, blood bags, tubes and dialysis equipments. Dibutyl phthalate (DBP) is widely used in many cosmetic products including perfume, lotion, and nail polish putting women to a higher health-hazard risk category. Ester phthalates family, monoethyl phthalate (MEP), monobutyl phthalate (MBP), monobenzyl phthalate (MBzP), and mono(2-ethylhexyl) phthalate (MEHP) have been detected in urine specimens, and their diester parent compounds in house dust samples, from pregnant and lactating women living in New York City [1]. Breast milk has been reported to contain phthalate metabolite monoesters in samples from Denmark/Finland [2], Sweden [3] and Italy [4].

Laboratory studies have shown that the toxicity of ester phthalates is caused by their metabolite, MAA. Gavage and intraperitoneal (i. p.) administration of MAA in rats resulted in their physical, reproductive, skeletal and hematopoietic abnormalities [5], [6] and it was shown to be a proximal teratogenic metabolite of 2-methoxyethanol (ME) and 2-Dimethoxyethyl phthalate (DMEP) [5], [7]. The toxicity of MAA has been shown to be mediated by multiple signaling pathways including oxidative stress, apoptosis, upregulation of heat shock proteins and deregulation of kinases, inhibition of histone deacetylase and disruption of estrogen receptor-mediated signaling [7]–[18]. In view of the extensive industrialization of present day lifestyle, there is a substantial urgency for molecular investigation to resolve the mechanism(s) of MAA toxicity and to invent novel ways and reagents to overcome its deleterious effects on human health.

In the present study, we report that a phytochemical, withanone, extracted from leaves of Ashwagandha protects human normal cells against the toxicity of MAA. We provide the very first molecular evidence showing the anti-toxic effects of withanone in normal human cells and propose its use as a MAA-antidote.

## Results and Discussion

### MAA causes senescence like growth arrest of normal human fibroblasts

**Figure 1:**
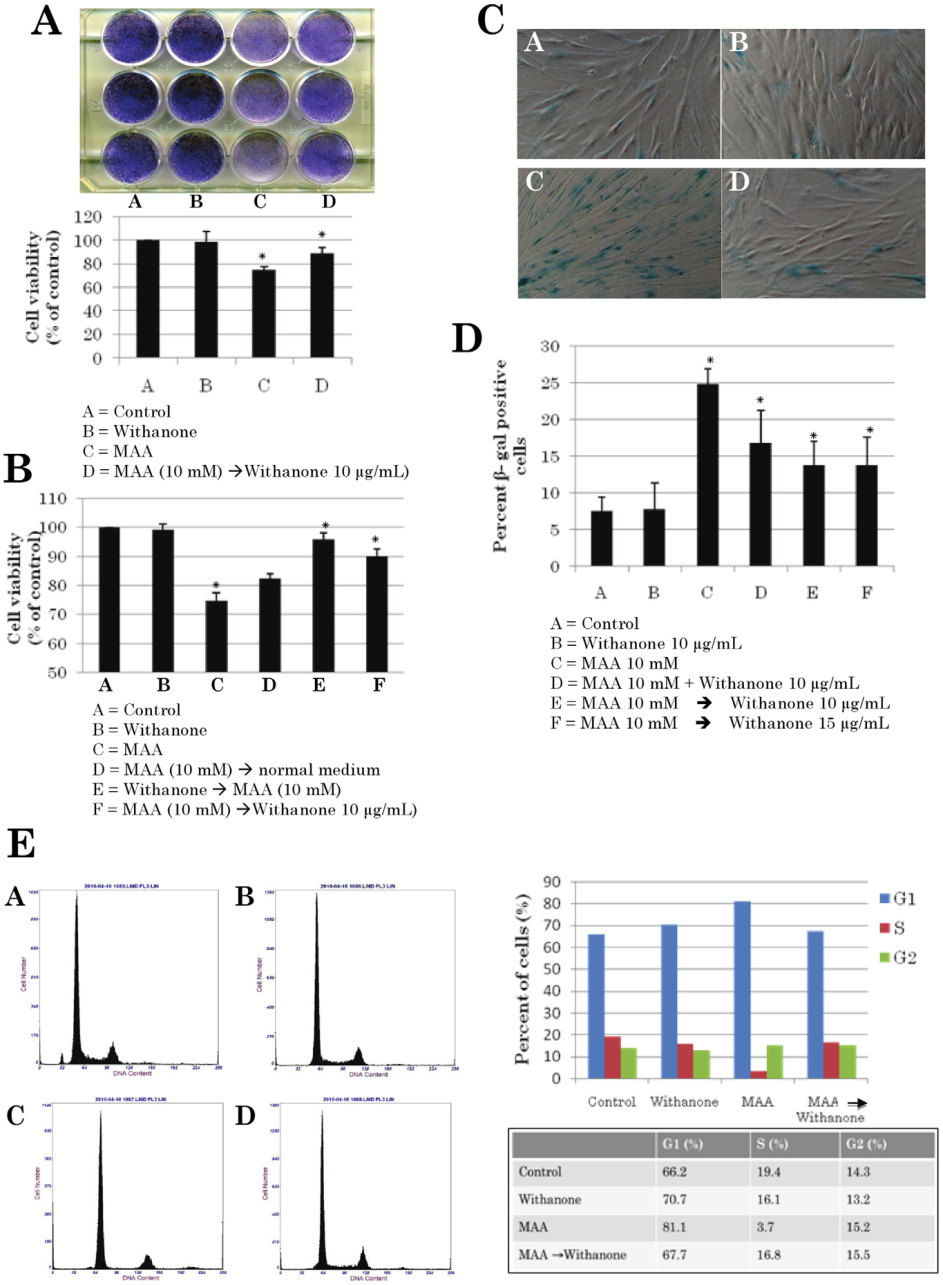
MAA-induced premature senescence in normal human cells. *A*, Crystal violet staining of normal human (TIG-1) fibroblasts treated with withanone (B), MAA (10 mM, 24 h)(C) and MAA followed by recovery in withanone-supplemented medium (MAA→withanone) (D). The difference between A & C and C & D were statistically significant (*P<0.05). *B*, Viability of cells treated with withanone (B), MAA (C), MAA followed by recovery either in normal medium (D) or in withanone-supplemented medium (F). Cells were also treated with withanone before MAA treatment (E). The difference between A & C, D & E and D & F were statistically significant (*P<0.05). *C*, Senescence-associated β-gal staining in TIG-1 cells treated with withanone (B), MAA (C) and MAA→withanone (D) treated cells. MAA treated cells showed increase in β-gal positive cells. The number of β-gal positive cells decreased when recovered in withanone-supplemented medium. Quantitation of β-gal positive cells from three independent experiments is shown in *D*. The differences between A & C, D & E and D & F were statistically significant (*P<0.05). *E*, Cell cycle distribution of control (A), withanone (B), MAA (C) and MAA→withanone (D) cells is shown. MAA led to arrest of cells in G1 cell cycle phase but was normalized when transferred to withanone-supplemented medium.

Normal human fibroblasts were cultured in the control medium for 24 h. Cells were then treated either with withanone- or MAA-supplemented medium for 48 h followed by recovery by placing them in either control normal medium or withanone-supplemented medium, respectively, for the next 24–48 h. As shown in Figure 1A, the treatment with MAA led to decrease in relative density of cells as detected by crystal violet DNA staining. Cell viability analysis by Alamar Blue®-cell proliferation assay revealed less number of viable cells in MAA-treated cultures. Cells cultured in the presence of withanone recovered from MAA-induced growth arrest and decline in viability. Of note, withanone treatment either before (Figure 1B–E) or after (Figure 1B–F) the exposure to MAA was effective in recovering the cells from MAA-induced cyto-toxicity. We next performed senescence-associated β-galactosidase staining assay in control and MAA-treated cells. Whereas MAA caused significant increase in senescence-associated β-galactosidase staining, the number of cells showing senescence like growth arrest (Figures 1C and 1D) declined in the presence of withanone suggesting that the MAA-induced cellular damage caused premature senescence in normal fibroblasts, and withanone protected the cells from such deteriorative phenotype. In order to get further insights to the kind of growth arrest induced by MAA, we performed cell cycle analysis on the control and treated cells. As shown in Figure 1E, MAA-treated cultures showed significant increase in number of cells in G1 stage of the cell cycle and decrease in number of cells in S phase. Subsequent recovery of MAA-treated cells in withanone-supplemented culture medium reinitiated the cell cycle progression and entry into S phase (Figure 1E).

### Molecular pathways involved in MAA-induced premature senescence in normal human fibroblasts

**Figure 2:**
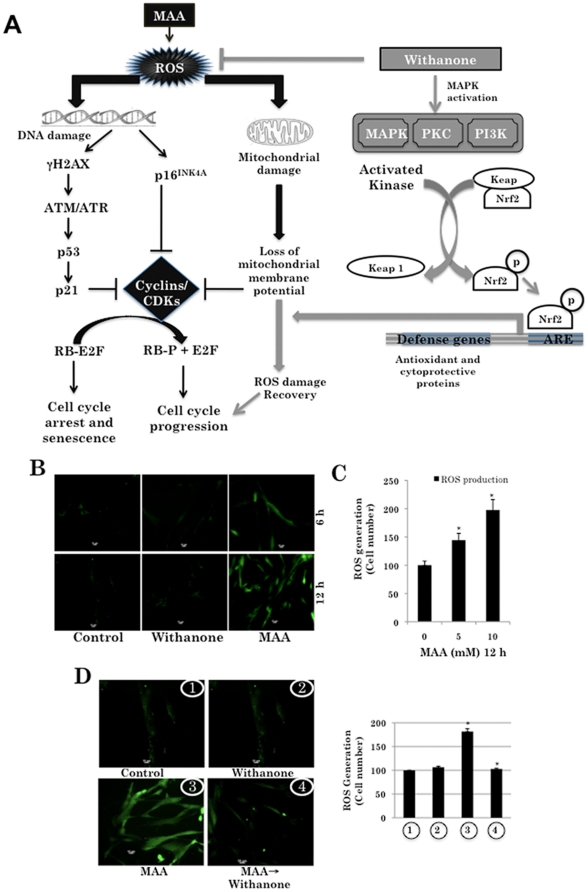
MAA-induced ROS generation in normal human cells. *A*, Schematic presentation of the pathways predicted to be involved in MAA-induced growth arrest/senescence of normal cells and its recovery by the addition of withanone. *B*, MAA-induced ROS generation in normal human cells as early as 6–12 h of treatment time. *C*, ROS level in cells treated with 10 mM MAA for 12 h was double compared to the control. Increase in ROS generation with 5 mM and 10 mM MAA were statistically significant (*P<0.05). *D*, Cells treated with MAA but recovered in withanone-supplemented medium showed reduction in ROS as compared to the control that were recovered in normal medium. The MAA-induced increase and withanone-induced recovery in ROS generation were statistically significant (*P<0.05).

In order to understand the molecular mechanism of MAA-induced growth arrest of normal human fibroblasts, we hypothesized that MAA may induce ROS resulting in oxidative damage to DNA and mitochondria, and that in turn induce DNA damage signaling and loss of mitochondrial membrane potential culminating in growth arrest of cells by pathways shown in Figure 2A. In order to test this hypothesis, we examined whether MAA caused ROS generation in cells. As shown in Figure 2B, cells treated with MAA for 6 and 12 h showed ROS generation, the amount of which was doubled in cells treated with 10 mM MAA for 12 h (Figure 2C). Furthermore, we found that while the cells treated with MAA showed ROS induction, the recovery treatment with withanone caused significant reduction in ROS indicating the involvement of ROS pathway in the process (Figures 2D and 2A).

### Involvement of ROS and DNA damage response in MAA-induced premature senescence of normal human fibroblasts and its recovery by withanone

**Figure 3:**
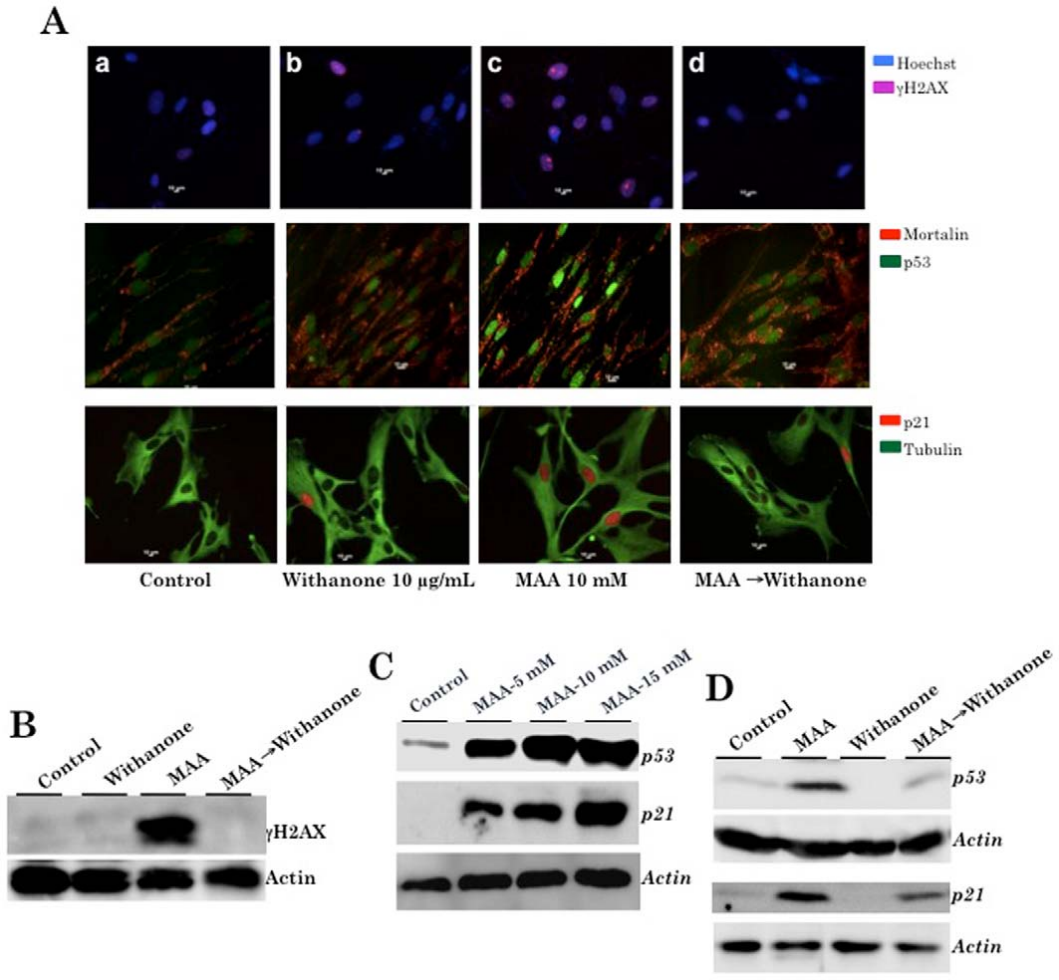
MAA-induced DNA damage and elevated level of p53-p21 expression in normal human cells. *A*, TIG-1 cells treated with withanone, MAA and MAA→withanone were immunostained for DNA damage and growth arrest marker proteins, γH2AX, p53 and p21. MAA-treated cells showed increase in all the three proteins. MAA→withanone treated cells did not show increase in any of these three proteins. *B*, Western blotting for γH2AX showing increase in MAA, but not in MAA→withanone treated cells. *C*, Western blotting for p53 and p21 in control and MAA treated cells. *D*, Western blotting for p53 and p21 in control, MAA, withanone and MAA→withanone treated cells. Actin was used as a loading control.

We next examined the above hypothesis by undertaking the expression analysis of molecules involved in ROS-induced DNA damage signaling and growth arrest of cells. As shown in Figure 3A, MAA caused an induction of DNA damage as examined by γH2AX focus formation. Noticeably, recovery of cells in withanone-supplemented medium resulted in the reduction of γH2AX expression (Figures 3A and 3B). These data demonstrated that the DNA damage caused by MAA was rectified by the addition of withanone. We next analyzed p53 and p21^WAF1^ (the two key molecular mediators of DNA damage response and senescence pathway) expression in MAA-treated and withanone-recovered cells. As shown in Figure 3C, p53 and p21 were induced in MAA-treated cells in a dose-dependent manner. Furthermore, MAA-treated and withanone-recovered cells showed considerable normalization of both p53 and p21^WAF1^ (Figures 3A and 3D). These data demonstrated that the MAA-induced DNA damage and growth arrest of normal cells were blocked by withanone.

**Figure 4:**
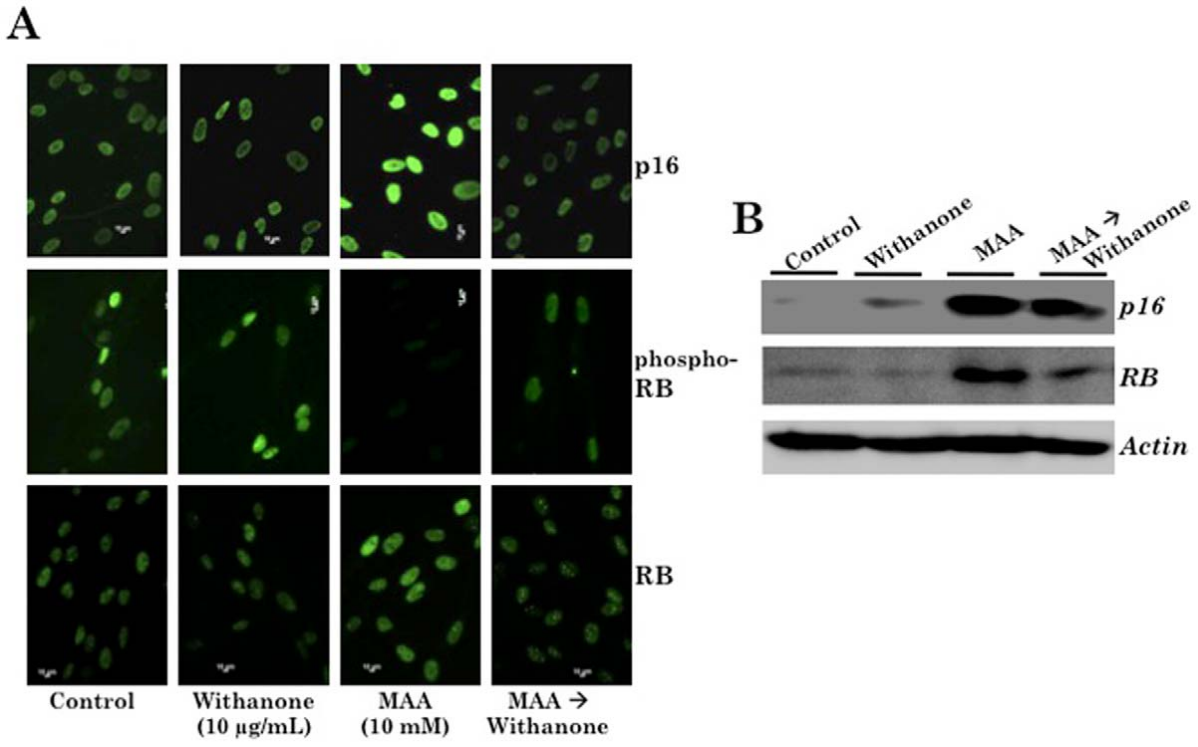
MAA-induced p16-RB expression in normal human cells. *A*, Cells treated with withanone, MAA and MAA→withanone were immunstained for p16 and RB. MAA-treated cells showed increase in p16 and RB. The level of phospho-RB decreased in MAA-treated cells. MAA→withanone treated cells did not show increase in either p16 or RB. Phospho-RB level was comparable to the control and withanone-treated cells. *B*, Western blotting for p16 and RB in control, withanone, MAA and MAA→withanone treated TIG-1 cells. Actin was used as a loading control.

We next examined p16^INK4A^-RB pathway, another major tumor suppressor pathway involved in senescence and has been shown to be upregulated in response to accumulation of DNA damage and replicative senescence of normal human fibroblasts. As shown in Figures 4A and 4B, p16^INK4A^ was upregulated in MAA-treated cells and its downstream effector RB (Figure 2A) was unphosphorylated (Figure 4A) leading to growth arrest of cells. These were consistent with the cell cycle, cell viability and p53-p21^WAF-1^ status data as described above. Of note, in contrast to the decrease in phospho-RB (Figure 4A), total RB expression was increased in MAA-treated cells (Figures 4A and 4B). Furthermore, consistent with the recovery from growth arrest, MAA-treated and withanone-recovered cells showed decrease in p16^INK4A^ (p16), recovery of phospho-RB and decrease in total RB (Figures 4A and 4B). These data revealed that the MAA-induced toxicity to normal cells involve p16^INK4A^-RB pathway and the treatment with withanone leads to their recovery as hypothesized in our model (Figure 2A).

### Involvement of ROS and mitochondrial damage during MAA-induced premature senescence of normal human fibroblasts and its recovery by withanone

**Figure 5:**
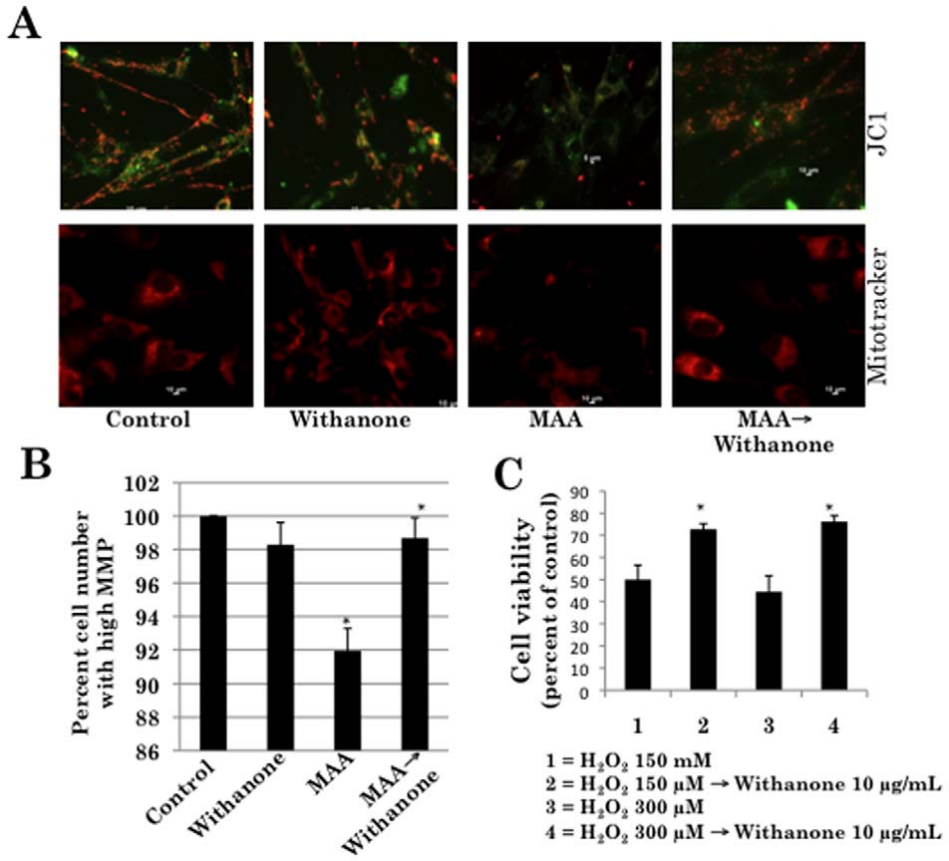
MAA-induced loss in mitochondrial membrane potential (MMP) in normal human cells. *A*, Cells treated with withanone, MAA and MAA→withanone were immunostained for JC1 and mitotracker. MAA-treated cells showed loss of MMP (loss of red staining). MAA→withanone treated cells showed mitochondrial membrane potential comparable to that of the control cells. *B*, Quantitation of cells showing red JC1 staining indicative of high mitochondrial membrane potential. MAA treatment caused decrease in the number of cells with high MMP and the recovery in the withanone-supplemented medium resulted in an increase in the number of cells with high MMP (*P<0.05). *C*, Cell viability analysis of the control, H_2_O_2_ and H_2_O_2_→withanone treated cells showing the recovery of cells. Cell viability was increased (statistical significance, *P<0.05) when they were recovered from the oxidative stress by incubation in the withanone-supplemented medium.

We next examined whether the ROS generated by MAA cause mitochondrial damage by analyzing mitochondrial membrane potential. We found that the cells when treated with MAA show loss of membrane potential as indicated by loss of both JC1-red and mitotracker staining. Most significantly, MAA-treated and withanone-recovered cells exhibited recovery of both JC-1 red and mitotracker staining suggesting that the mitochondrial membrane potential of these cells was comparable to the normal untreated cells (Figures 5A and 5B). Furthermore, to test whether the treatment with withanone protected the normal cells against mitochondrial damage, we also exposed these cells to oxidative damage by treating with H_2_O_2_. As shown in Figure 5C, cell viability was significantly improved when H_2_O_2_-treated cells were recovered in withanone-supplemented medium demonstrating that whereas MAA caused ROS generation, DNA and mitochondrial damage, withanone protected the cells against such damage by suppressing ROS generation as hypothesized in our model (Figure 2A).

### Protection against ROS and MAA-induced premature senescence of normal human fibroblasts involve an induction of Nrf2-ARE signaling

**Figure 6:**
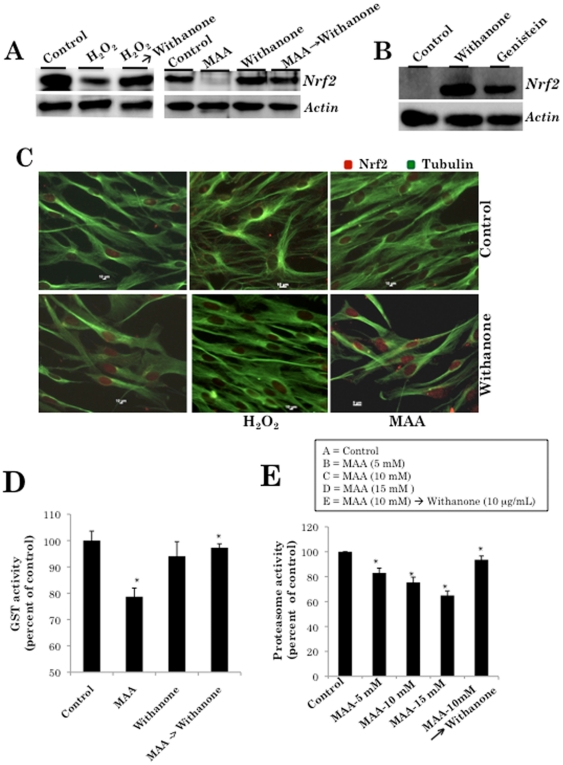
MAA-induced Nrf2 pathway in normal human cells. *A*, Cells treated with either H_2_O_2_ or MAA showed reduction in Nrf2. Cells treated with H_2_O_2_→withanone or MAA→withanone showed increase in Nrf2. *B*, Induction of Nrf2 by withanone was stronger than genistein, a known inducer of Nrf2 pathway. *C*, Nuclear translocation of Nrf2 in withanone, H_2_O_2_→withanone or MAA→withanone cells was observed by immunostaining with Nrf2 specific antibody. *D*, MAA treated cells showed loss in GST activity and its recovery was observed in MAA→withanone cells (*P<0.05). *E*, Proteasomal activity was decreased in MAA treated cells but recovered in MAA→withanone cells (*P<0.05).

Keap1-Nrf2-ARE signaling plays a significant role in protecting cells from endogenous and exogenous stresses. Under normal conditions, the transcription factor Nrf2, a primary transcription factor required for the induction of a battery of phase II detoxification genes through activation of antioxidant response element (ARE), interacts with the actin-anchored protein Keap1 and is largely localized in the cytoplasm resulting in the low basal expression of Nrf2-regulated genes. However, upon recognition of chemical signals imparted by oxidative and electrophilic molecules that cause activation of MAP kinase signaling, Nrf2 is phosphorylated, released from Keap1, escapes proteasomal degradation, translocates to the nucleus, and transactivates the expression of several antioxidant and cytoprotective genes that increase resistance to oxidative stress and mitochondrial dysfunction leading to enhance cell survival (Figure 2A) [19]. We first examined Nrf2 in normal human fibroblasts treated with oxidative stress (H_2_O_2_) and during recovery phase in withanone-supplemented medium. As expected, normal cells treated with cytotoxic doses of hydrogen peroxide and MAA showed reduction in Nrf2. However, the cells treated with withanone resulted in an induction of Nrf2 at much higher level than by genistein, a known inducer of Nrf2 pathway (Figures 6A and 6B). Whereas MAA-treated cells showed suppression of Nrf2, the MAA-treatment followed by addition of withanone showed recovery through induction of Nrf2. As shown in Figure 6C, withanone-recovered cells showed nuclear translocation of Nrf2, associated with expression of ARE-regulated gene glutathione *S*-transferase (GST) (Figure 6D). There was a loss of 25–30% in GST activity in MAA-treated cells; withanone-recovered cells showed 15–20% GST recovery. We also examined the proteasomal activity in MAA-treated and withanone added recovered cells. Withanone caused recovery of the MAA-induced decrease in the proteasomal activity (Figure 6E).

The present day life heavily depends on industrial products and hence it is a major challenge to protect human health against the toxic effects of industrial chemicals. Genetic and functional toxicity of MAA, a major metabolite of ester phthalates, has been shown in a variety of *in vitro* and *in vivo* model studies [9], [16], [20]–[23]. It has been shown to cause change in gene expression pattern [8], cell environment [22] and mitochondrial function [24]. We had previously shown that Ashwagandha leaf extract (i-Extract) and withanone, a major constituent in the leaf, cause selective killing of cancer cells [25], [26]. Normal human fibroblasts were benefited by treatment with i-Extract; these showed increase in lifespan, reduction in the molecular damage and protection against oxidative stress [27], [28]. At this end, we investigated whether MAA-induced damage to normal human cells in culture could be recovered by the treatment with withanone. As shown above, we found that the MAA caused ROS-mediated DNA and mitochondrial damage to normal human cells. The cells showed significant recovery from such damages when subsequently treated with withanone (Figures 2, 3, 4, 5). Furthermore, withanone stimulated the Nrf2 signaling resulting in the nuclear translocation of Nrf2 and an increase in GST activity (Figure 6D) suggesting the increase in cellular defense mechanism to overcome the oxidative stress. Proteasomal function was also increased in cells that were treated with withanone (Figure 6E). Taken together, these data suggested that the cells treated with withanone could be protected against MAA-toxicity by multiple mechanisms including reduction in the production of ROS, subsequent damage at DNA and mitochondrial level, and induction of cellular defense machinery including Nrf2 signaling and proteasomal degradation. As a result of these molecular effects of withanone, human normal cells were protected against MAA-induced toxicity indicated by growth arrest and premature senescence (Figure 1). These data suggest that withanone is a strong candidate for health adjuvant and could be recruited in multiple industrial products including cosmetics, toiletry, and medical products containing ester phthalates that are toxic and threat to human health.

## Materials and Methods

### Cell culture, treatments and viability assay

Normal human fibroblasts (TIG-1) were obtained from the Japanese Collection of Research Bio-resources (JCRB, Japan) and grown at 37°C in a 5% CO_2_ incubator in low glucose Dulbecco's modified Eagle's minimal (DMEM; Gibco BRL, Grand Island, NY) essential medium supplemented with 10% fetal bovine serum. Cells (at about 50% confluency) were treated with 10 mM Methoxyacetic Acid (MAA) (WAKO Chemicals, Japan) for 24 h followed by culture in a medium-containing withanone 10 µg/mL for 24 h for recovery treatments. For pretreatment, withanone was added to the culture medium 24 h before the addition of MAA and was continued during the MAA-treatement. Control (MAA treatment for 24 h followed by recovery in normal culture medium) and treated cells were analyzed as described below.

Cell viability was examined using Alamar Blue®-cell proliferation assay kit (Invitrogen) following manufacturer's instructions. Briefly, cells (3×10^3^ cells) were plated in 96-well plates. After 24 h of incubation, cells were treated with MAA (10 mM, for 24 h) followed by culture in a fresh DMEM medium-supplemented with Withanone (10 µg/mL) for 24 h. Cells were then incubated with Alamar Blue (10% in DMEM) for 1 h. Change in Alamar Blue color from blue nonfluorescent to red fluorescent (proportional to the living and metabolically active cells) was recorded at 450 nm (absorbance is monitored at 570 nm and 600 nm) in a microplate reader. Cell viability was also assayed by Crystal violet staining. Control and treated cells were washed with PBS and fixed in 1% paraformaldehyde (15 mins at room temperature). After washing twice with PBS, the cells were incubated with Crystal violet dye (0.5% in H_2_O) at room temperature overnight. Cells were washed with H_2_O and plates were photographed with image scanner (EPSON GT-9800F).

### Immunostaining

Immunostaining for the indicated proteins was performed as described earlier [25]. Cells were grown on glass coverslips placed in 12-well culture dishes. At the end of treatment, cells were washed with cold phosphate-buffered saline (PBS) and fixed with a pre-chilled methanol/acetone (1/1, v/v) mixture for 5 min on ice. Fixed cells were washed thrice with PBS, permeabilized with 0.2% Triton X-100 in PBS for 10 min, and blocked with 2% bovine serum albumin (BSA) in PBS for 20 min. Cells were stained with antibodies against p53 (DO-1, Santa Cruz Biotech), p21^WAF-1^ (C-19, Santa Cruz Biotech), p16^INK4A^ (Ab-F12, Santa Cruz Biotech), RB (Ab 4H1, Cell Signaling Technology), phosphor-Rb (Ser 780, Cell Signaling Technology), Nrf2 (H-300, Santa Cruz Biotech), γH2AX (Biolegend) and β-tubulin (Sigma). Immunostaining was visualized by secondary staining with Alexa-594-conjugated goat anti-rabbit and Alexa 488-conjugated goat anti-mouse (Molecular Probes, Eugene, OR) antibodies. After washing with PBS-TX (0.1% Triton X-100 in PBS thrice) and PBS (once), cells were overlaid with FA mounting fluid (VMRD, Inc). The cells were examined under a microscope (Axiovert 200M; Carl Zeiss, Thornwood, NY) attached to an AxioCam MRm monochrome charge-coupled device camera (Carl Zeiss). The images were processed with AxioVision software (version 4.4; Carl Zeiss).

For **γ**H_2_AX immunostaining, cells were permeabilized with KCM solution (120 mM KCl, 20 mM NaCl, 10 mM Tris pH 7.5, 0.1% Triton) and blocked with solution containing 2% BSA, 0.2% Gelatin, 150 mM NaCl, 0.1% Triton X-100, 20 mM Tris pH 7.5, 0.1% Sodium Azide in MilliQ water for 30 mins.

### Western blotting

Expression level of indicated proteins was examined by Western blotting. Control and treated cells were harvested by trypsin, washed and lysed in buffer containing 1% Nonidet P-40 in TBS (20 mM Tris, 150 mM NaCl, pH 7.4)-supplemented with 10 µg/ml of a protease inhibitor mixture (Roche). The whole cell lysate was centrifuged at 12,000 rpm for 10–20 min at 4°C and supernatant (10–20 µg) was resolved on SDS-polyacrylamide gel electrophoresis under reducing conditions. The gel was transferred onto Immobilon-P membranes (Millipore) using a semi-dry transfer apparatus (ATTO, Japan). Membranes were probed with antibodies against p53 (DO-1, Santa Cruz Biotech), p21^WAF-1^ (C-19, Santa Cruz Biotech), p16^INK4A^ (AbF12, Santa Cruz Biotech), Rb (Ab4H-1, Cell Singaling Technology), Nrf2 (H-300, Santa Cruz Biotech), γH2AX (Biolegend) and actin (Chemicon). After washing with TBS-T, the membrane was incubated with horseradish peroxidase-conjugated secondary antibody for 30 min. The membrane was washed thrice in TBS-T and once in TBS, then subjected to an enhanced chemiluminescence (ECL)-mediated visualization (Amersham Biosciences) using Lumino Image Analyzer equipped with charge-coupled device (CCD) camera (LAS3000-mini, Fuji Film).

### Cell cycle analysis

The cells (about 50% confluent) were treated with MAA for 24 h followed by incubation in either control medium or in withanone-supplemented medium for another 24 h for recovery and then harvested by trypsin. Cells pellets were washed with cold PBS and fixed in 70% ethanol at 4°C for 12 h. The fixed cells were centrifuged at 2000 rpm for 10 min, washed with cold PBS twice and then re-suspended in 0.25 ml PBS. The cells suspensions were treated with RNAse A (5 µl, 1 mg/ml at 37°C for 1 h) to remove RNA followed by incubation with Propidium Iodide (PI, 10 µl, 1 mg/ml) for 30 min in dark. PI-stained cells were subjected to cell cycle analysis using Coulter Epics XL™ Flow Cytometer (Beckman).

### Senescence-associated β-galactosidase assay

Control and treated cells were subjected to senescence-associated β-galactosidase (SA-β-gal) staining using the standard protocol. In brief, cells were first treated with MAA and then incubated in either control medium or in withanone-supplemented medium for recovery. The cells were washed with PBS, fixed in 2% formaldehyde/0.2% glutaraldehyde in PBS for 10 min, and washed again with PBS, followed by an overnight incubation in staining solution (citric acid/phosphate buffer, pH 6.0, 5 mM K_3_Fe(CN)_6_, 5 mM K_4_Fe(CN)_6_, 2 mM MgCl_2_, 150 mM NaCl, supplemented with 1 mg/ml of 5-bromo-4-chloro-3-indolyl-β-D-galactopyranoside (X-gal) at 37°C. Stained cells were examined under the microscope and photodocumented with a NIKON camera.

### Proteasome activity assay

Control and treated cells were harvested with cell scraper and assayed for proteasome activity using Proteasome Activity Assay kit (Chemicon). The assay is based on the detection of the fluorophore 7-Amino-4-methylcoumarin (AMC) after cleavage from the labeled substrate LLVY-AMC. The free AMC fluorescence was quantitated using a 380/460 nm filter set in a fluorometer.

### Glutathione-S-transferase assay

Control and treated cells were subjected for analysis using Glutahione-S-transferase Assay Kit (Cayman Chemical Company) following manufacturer's instructions. The cells (3×10^4^) were seeded in 100-mm plate and treated with MAA for 24 h followed by recovery either in normal or withanone-supplemented medium for 24 h. Cells were then washed with cold PBS, scraped and centrifuged at 2000× *g* for 10 min at 4°C. Cell pellets were sonicated in cold 1–2 ml of cold buffer (100 mM potassium phosphate, pH 7.0, containing 2 mM EDTA), centrifuged at 10,000× *g* for 15 min at 4°C. The supernatant was subjected to assay. Reduced glutathione (GSH) content in supernatant was measured colorimetrically at 340 nm using plate reader.

### Detection of reactive oxygen species

The reactive oxygen species were detected by fluorescent staining using the Image-iT™ LIVE Green Reactive Oxygen Species (ROS) Detection Kit (Molecular Probes Inc, USA). Cells were grown on glass coverslips placed in 12-well plate and were treated as described above. Cells were fixed and then stained for ROS by procedure recommended by the manufacturers.

### JC1 staining and mitochondrial damage

Control and treated cells were incubated in JC-1 (Molecular probes) 10 µg/ml dye containing media at 37°C for 10 min. The JC-1 labeled cells were detected by fluorescent microscopy. Cells were also incubated with MitoTracker Deep Red (150 nM) for 30 min and examined under fluorescence microscope (Carl Zeiss).

### Statistical analysis

Statistical significance of the data (viability, SA-ß-gal assay, ROS generation, mitochondrial membrane potential, GST and proteasomal activity) was calculated from three independent experiments using One-way ANOVA and Baferroni's test. The values were considered significant for P<0.05.

## References

[1] Adibi JJ, Whyatt RM, Williams PL, Calafat AM, Camann D (2008). Characterization of phthalate exposure among pregnant women assessed by repeat air and urine samples.. Environ Health Perspect.

[2] Main KM, Mortensen GK, Kaleva MM, Boisen KA, Damgaard IN (2006). Human breast milk contamination with phthalates and alterations of endogenous reproductive hormones in infants three months of age.. Environ Health Perspect.

[3] Högberg J, Hanberg A, Berglund M, Skerfving S, Remberger M (2008). Phthalate diesters and their metabolites in human breast milk, blood or serum, and urine as biomarkers of exposure in vulnerable populations.. Environ Health Perspect.

[4] Latini G, Wittassek M, Del Vecchio A, Presta G, De Felice C (2009). Lactational exposure to phthalates in Southern Italy.. Environ Int.

[5] Brown NA, Holt D, Webb M (1984). The teratogenicity of methoxyacetic acid in the rat.. Toxicol Lett.

[6] Miller RR, Carreon RE, Young JT, McKenna MJ (1982). Toxicity of methoxyacetic acid in rats.. Fundam Appl Toxicol.

[7] Bagchi G, Waxman DJ (2008). Toxicity of ethylene glycol monomethyl ether: impact on testicular gene expression.. Int J Androl.

[8] Bagchi G, Zhang Y, Waxman DJ (2010). Impact of methoxyacetic acid on mouse Leydig cell gene expression.. Reprod Biol Endocrinol.

[9] Henley DV, Mueller S, Korach KS (2009). The short-chain fatty acid methoxyacetic acid disrupts endogenous estrogen receptor-alpha-mediated signaling.. Environ Health Perspect.

[10] Jindo T, Wine RN, Li LH, Chapin RE (2001). Protein kinase activity is central to rat germ cell apoptosis induced by methoxyacetic acid.. Toxicol Pathol.

[11] Rao AV, Shaha C (2002). N-acetylcysteine prevents MAA induced male germ cell apoptosis: role of glutathione and cytochrome c.. FEBS Lett.

[12] Ruyani A, Sudarwati S, Sutasurya LA, Sumarsono SH, Kim DJ (2005). A teratoproteomics analysis: heat shock protein 70 is upregulated in mouse forelimb bud by methoxyacetic acid treatment.. Birth Defects Res A Clin Mol Teratol.

[13] Scofield EH, Henderson WM, Funk AB, Anderson GL, Smith MA (2006). Diethylene glycol monomethyl ether, ethylene glycol monomethyl ether and the metabolite, 2-methoxyacetic acid affect in vitro chondrogenesis.. Reprod Toxicol.

[14] Syed V, Hecht NB (1998). Rat pachytene spermatocytes down-regulate a polo-like kinase and up-regulate a thiol-specific antioxidant protein, whereas sertoli cells down-regulate a phosphodiesterase and up-regulate an oxidative stress protein after exposure to methoxyethanol and methoxyacetic acid.. Endocrinology.

[15] Takagi A, Yamada T, Hayashi K, Nakade Y, Kojima T (2002). Involvement of caspase 3 mediated apoptosis in hematopoietic cytotoxicity of metabolites of ethylene glycol monomethyl ether.. Ind Health.

[16] Louisse J, Bai Y, Verwei M, van de Sandt JJ, Blaauboer BJ (2010). Decrease of intracellular pH as possible mechanism of embryotoxicity of glycol ether alkoxyacetic acid metabolites.. Toxicol Appl Pharmacol.

[17] de Jong E, Louisse J, Verwei M, Blaauboer BJ, van de Sandt JJ (2009). Relative developmental toxicity of glycol ether alkoxy acid metabolites in the embryonic stem cell test as compared with the in vivo potency of their parent compounds.. Toxicol Sci.

[18] Elkin ND, Piner JA, Sharpe RM (2010). Toxicant-induced leakage of germ cell-specific proteins from seminiferous tubules in the rat: relationship to blood-testis barrier integrity and prospects for biomonitoring.. Toxicol Sci.

[19] Yates MS, Kensler TW (2007). Chemopreventive promise of targeting the Nrf2 pathway.. Drug News Perspect.

[20] Davis BJ, Almekinder JL, Flagler N, Travlos G, Wilson R (1997). Ovarian luteal cell toxicity of ethylene glycol monomethyl ether and methoxyacetic acid in vivo and in vitro.. Toxicol Appl Pharmacol.

[21] Bagchi G, Hurst CH, Waxman DJ (2009). Interactions of methoxyacetic acid with androgen receptor.. Toxicol Appl Pharmacol.

[22] Ruyani A, Sudarwati S, Sutasurya LA, Sumarsono SH, Gloe T (2003). The laminin binding protein p40 is involved in inducing limb abnormality of mouse fetuses as the effects of methoxyacetic acid treatment.. Toxicol Sci.

[23] Welsch F (2005). The mechanism of ethylene glycol ether reproductive and developmental toxicity and evidence for adverse effects in humans.. Toxicol Lett.

[24] Beattie PJ, Brabec MJ (1986). Methoxyacetic acid and ethoxyacetic acid inhibit mitochondrial function in vitro.. J Biochem Toxicol.

[25] Widodo N, Kaur K, Shrestha BG, Takagi Y, Ishii T (2007). Selective killing of cancer cells by leaf extract of Ashwagandha: identification of a tumor-inhibitory factor and the first molecular insights to its effect.. Clin Cancer Res.

[26] Widodo N, Takagi Y, Shrestha BG, Ishii T, Kaul SC (2008). Selective killing of cancer cells by leaf extract of Ashwagandha: Components, activity and pathway analyses.. Cancer Lett.

[27] Widodo N, Shah N, Priyandoko D, Ishii T, Kaul SC (2009). Deceleration of senescence in normal human fibroblasts by withanone extracted from ashwagandha leaves.. J Gerontol A Biol Sci Med Sci.

[28] Widodo N, Priyandoko D, Shah N, Wadhwa R, Kaul SC (2010). Selective killing of cancer cells by Ashwagandha leaf extract and its component withanone involves ROS signaling.. PLoS One.

